# Influence of Early Bladder Imaging in Experimental Rabbits on the Quantitative Determination of Glomerular Filtration Rate by the Gates Method

**DOI:** 10.1155/2020/8848189

**Published:** 2020-11-26

**Authors:** Changyin Wang, Shun Li, Chun Gao, Wasili Maimaiti, Qisheng Yang, Linglong Jiang

**Affiliations:** ^1^Department of Nuclear Medicine, Zhongnan Hospital of Wuhan University, Wuhan 430071, China; ^2^Second Clinical Faculty, Medical School of Wuhan University, Wuhan 430071, China; ^3^Surgical Department of Emergency Center, Zhongnan Hospital of Wuhan University, Wuhan 430071, China

## Abstract

**Objective:**

To investigate the influence of early bladder imaging (EBI) in experimental rabbits on the quantitative calculation of glomerular filtration rate (GFR) by the Gates method.

**Methods:**

We retrospectively analyzed the data of dynamic renal scintigraphy (DRS) in experimental rabbits. We calculated renal uptake during minutes 1-2 and 2-3 by correcting bladder radioactivity and computed the split GFR by renal uptake. Then, the EBI and GFR between 1-2 min and 2-3 min were compared, respectively.

**Results:**

The EBI proportion (57.3%) at 2-3 min of DRS was higher than that (8.5%) at 1-2 min (*P* < 0.05). The correlations between the 1-2 min and 2-3 min uptake rates of unobstructed kidneys after correction (*r* = 0.952‐0.979) were higher than those before correction (*r* = 0.859‐0.936). However, the correlation between the two in obstructed kidneys was not improved (*r*_before_ = 0.967 versus *r*_after_ = 0.968). For unobstructed kidneys, the difference in GFR based on 2-3 min uptake between before and after correction was significant (*P* < 0.05), but not in obstructed kidneys (*P* > 0.05). For GFR based on 1-2 min uptake, the difference between before and after correction was not significant in obstructed or unobstructed kidneys (*P* > 0.05). Before correction, the GFR of unobstructed kidneys of 10.5% of the rabbits in the protein load test was lower than that in the baseline status, but not so after correction.

**Conclusion:**

The 2-3 min EBI on DRS has a significant influence on the GFR calculated by the Gates method in experimental rabbits. Controlling water intake or calculating the GFR by 1-2 min renal uptake helps to avoid the influence of EBI on GFR.

## 1. Introduction

The glomerular filtration rate (GFR) is an important parameter for the diagnosis of chronic kidney disease [1, 2]. Clinically, dynamic renal scintigraphy (DRS) is widely used to evaluate the renal function of patients. Gates established a method for quantitative calculation of GFR based on DRS [3–6]. The GFR calculated by the Gates method is a parameter that can be used to evaluate split renal function. This method is not only commonly used in humans but is also used to evaluate the renal function of experimental animals [7–9]. For humans, there are many factors that can affect the accuracy of GFR measured by the Gates method [10–13]. Many scholars have studied and improved this method to increase its reliability [14–18]. However, animals are different from humans. In animals, are there more influencing factors? This is a question that should be considered when the Gates method is applied in animals. The research on the applicability of this method in animals is very limited. When the Gates method is used to measure GFR, we need the renal depth and the body surface area. Clinically, the method estimating the renal depth and body surface area based on height and weight has been established in human subjects, but it is not necessarily suitable for animals. The renal depth of animals needs to be measured [8, 9], and animal GFR is often corrected by body weight [7–9], but not by body surface area. For animals, there are some different options for the time period in which renal uptake rate is calculated [8, 9, 19], which are different from those of humans. Kampa et al. [19] considered that it was most appropriate to calculate the GFR using the renal uptake rate at 30-120 sec after injecting a radiotracer; however, they did not analyze the extent of the influence on the results of measuring at other time periods. In order to explore the influence of bladder imaging time on the measurement of animal GFR, we observed and compared the appearance time of a radiotracer in the rabbit bladder, analyzed the degree of its influence on GFR, and reported them as follows.

## 2. Materials and Methods

### 2.1. Study Materials

This study was approved by the Animal Care and Use Committee of Wuhan University Center for Animal Experiments (AUP number: 2013110). All procedures and animal handlings were performed following the ethical guidelines for animal studies. The data of white rabbits that were submitted for DRS in our research in recent years were collected as research materials. Among them were 30 Japanese white rabbits, 8 weeks old, weighing 2.0-3.0 kg, male or female, and 15 New Zealand white rabbits, 8 weeks old, weighing 2.16-2.74 kg, with an average of 2.51 ± 0.17 kg, male. For the Japanese white rabbits, six were used as normal controls and 24 were made into right ureteral obstruction models by the ureteral casing method [20]. Twelve of them were finally relieved of obstruction, and 30 rabbits underwent 42 DRS examinations. The New Zealand white rabbits were also made into the right ureteral severe obstruction model by the ureteral casing method [21, 22]. A total of 198 DRS examinations in baseline status were performed at different times before operating and after obstruction, and 24 DRS examinations were performed for the protein load test. In a total of 264 examinations, 211 examinations with part or all of the bladder image in the field of view were selected for calculation and analysis.

### 2.2. Dynamic Renal Scintigraphy

The imaging instrument was a single-photon-emission computed tomography (SPECT) machine with a low-energy, high-resolution collimator (e.cam; Siemens product, Hoffman Estates, Illinois, USA). The ^99^Mo-^99m^Tc generator was supplied by Beijing Atomic High-Tech Co., Ltd. Diethylene triamine pentaacetate acid (DTPA) was provided by Beijing Xinke Sida Pharmaceutical Technology Co., Ltd. Rabbits were anesthetized by intraperitoneal injection [20–22]. After anesthesia, the rabbits were fixed on a thin board in the supine position. To avoid the influence of the thin board on quantitative results, the full syringe, the empty syringe, and the rabbits were placed on the same thin board for imaging. Imaging conditions of Japanese white rabbits were as follows: for full-syringe and empty-syringe imaging, the distance between the syringe and detector was 30 cm, the matrix used was128 × 128, acquisition magnification was 1.0, and acquisition time was 1 min; for the blood perfusion phase, the matrix used was128 × 128, acquisition magnification was 2.0, acquisition time was 4 sec/frame, and the total number of frames used was 15; for the renal parenchyma phase, the matrix used was128 × 128, acquisition magnification was 2.0, acquisition time was 60 sec/frame, and the total number of frames used was 20. Imaging conditions of New Zealand white rabbits were as follows: for full-syringe and empty-syringe imaging, the distance between the syringe and detector was 30 cm, the matrix used was256 × 256, acquisition magnification was 1.0, and acquisition time was 1 min; for the perfusion phase, the matrix used was256 × 256, acquisition magnification was 2.67, acquisition time was 3 sec/frame, and the total number of frames used was 20; for the renal parenchyma phase, the matrix used was256 × 256, acquisition magnification was 2.67, acquisition time was 10 sec/frame, and the total number of frames used was 30.

### 2.3. Study Methods

Early bladder imaging (EBI) is based on the fact that the radiotracer appears in the bladder of rabbits within 3 min after intravenously injecting imaging agents. We read the imaging data of all rabbits; observed the distribution of radiotracers in the bladder during 0-1 min, 1-2 min, and 2-3 min after injection; and calculated the percentage of scintigraphy examinations with EBI. The difference in the rate of EBI between 1-2 min and 2-3 min was compared. When EBI was not considered, the renal uptake rate with depth correction during 1-2 min and 2-3 min was calculated [4], and the correlation between them was analyzed. The net bladder radioactivity was calculated in EBI. For dual kidneys without any obstruction, the net bladder radioactivity was averagely distributed to the bilateral kidneys, and then the uptake rate of the bilateral kidneys was calculated. For rabbits with right severe ureteral obstruction, the radiotracer that presented early in the bladder might only be derived from the left kidney, so the net bladder radioactivity was all distributed to the left kidney, and the uptake rate of the bilateral kidneys was calculated separately. The correlation between the 2-3 min and 1-2 min renal uptake rates was analyzed when considering EBI. Then, the correlations in the renal uptake rate between considering and not considering EBI were compared. GFR was calculated by the Gates formula [4] using the data for 1-2 min and 2-3 min. The difference between renal GFR without bladder radiotracer correction and that with bladder radiotracer correction was compared. The difference in GFR between baseline status and during the protein load test was compared, and the influence of EBI on the result of the protein load test is discussed.

### 2.4. Statistical Analysis

SPSS software (version 22.0; IBM Corporation, Armonk, New York, USA) was used to process the data. The data are represented by scatter plots and the mean ± standard deviation. The chi-square test was used to compare the rates. The bladder radiotracer uptake rate and its contribution from renal GFR at 1-2 min vs. 2-3 min after injection were compared by the two-independent-samples *t*-test. The comparison between the GFR corrected and uncorrected by bladder radioactivity and the comparison between GFR at baseline and in the protein load test were performed with a paired *t*-test. The correlation between 1-2 min and 2-3 min renal uptake rate was analyzed by the bivariate Pearson correlation analysis.

## 3. Results

### 3.1. Comparison of EBI Rate in Experimental Rabbits after Injecting the Radiotracer

In the 211 examinations in which the experimental rabbits' bladders were in the field of view, the bladders of all animals were invisible during 0-1 min after injecting the radiotracer. During 1-2 min after injecting the radiotracer, the animals had EBI in 8.5% (18/211) of the DRS scans. During 2-3 min after injecting the radiotracer, the animals had EBI in 57.3% (121/211) of the examinations. The proportion of DRS scans in which the animals had EBI during 1-2 min was significantly lower than that during 2-3 min (*χ*^2^ = 113.8, *P* < 0.001). Thus, at 1-2min after injecting the radiotracer, EBI begins to appear in a very small number of rabbits, and at 2-3 min after injecting the radiotracer, the number of animals that had EBI rapidly increases.

### 3.2. Comparison of Bladder Radiotracer Uptake and Its Contribution from Renal Filtration in Experimental Rabbits

Table 1 shows that the bladder radiotracer uptake rate at 2-3 min after injection is higher than that at 1-2 min (*t* = 2.800, *P* = 0.006), and the proportion of the bladder radiotracer that came from renal filtration at 2-3 min is greater than that at 1-2 min (*P* < 0.05). These findings show that the bladder radiotracer uptake and its contribution from renal filtration are small at 1-2 min after injection, but they increase significantly at 2-3 min.

### 3.3. Correlation of Renal Uptake Rates between 1-2 Min and 2-3 Miner

**Figure 1:**
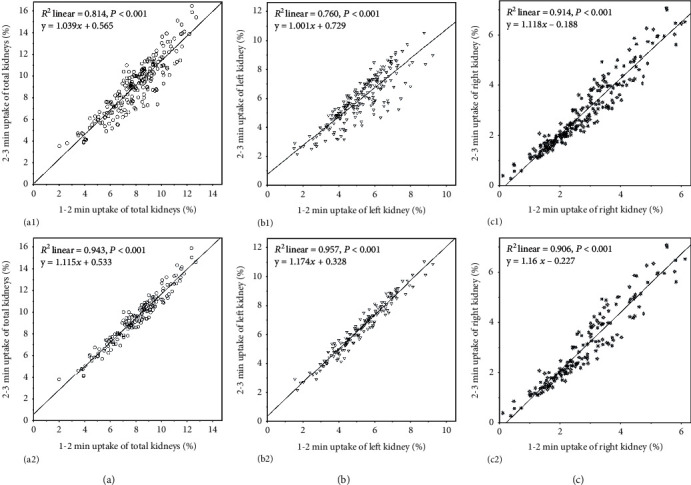
Linear regression results and scatter plots between 1-2 min and 2-3 min renal uptake rates. (a) Total kidney: (a1) the total renal uptake rate without correction by bladder radioactivity; (a2) the total renal uptake rate with correction by bladder radioactivity. (b) Left kidney: (b1) the left renal uptake rate without correction by bladder radioactivity; (b2) the left renal uptake rate with correction by bladder radioactivity. (c) Right kidney: (c1) the right renal uptake rate without correction by bladder radioactivity; (c2) the right renal uptake rate with correction by bladder radioactivity.

Table 2 shows that there is a positive correlation between the renal uptake rate at 1-2 min and that at 2-3 min (*P* < 0.001), whether the kidneys are hydronephrotic or not. When the kidney uptake was not corrected by the bladder radioactivity, the correlation and linearity between the uptake rate of obstructed kidneys at 1-2 min and that at 2-3 min were higher than the correlation and linearity of unobstructed kidneys (Table 2, Figures 1(b)1 and 1(c)1). For the kidney without ureteral obstruction, the correlation and linearity between the 1-2 min uptake rate corrected by bladder radioactivity and the corrected 2-3 min uptake rate were significantly improved in comparison with those of no correction (Table 2, Figures 1(b)1 and 1(b)2). For the kidney with ureteral obstruction, there was no significant improvement in the correlation or linearity between the corrected renal uptake rate at 1-2 min and that at 2-3 min by comparison with no correction (Table 2, Figures 1(c)1 and 1(c)2).

### 3.4. Comparison of Renal GFR Corrected by Bladder Radioactivity with Uncorrected GFR

Table 3 shows that for unobstructed kidneys, when the GFR was calculated by the 1-2 min renal uptake rate, the difference between the GFR corrected by the bladder radioactivity and the uncorrected GFR was not statistically significant (*P* > 0.05). When the GFR was calculated by the 2-3 min renal uptake rate, the GFR corrected by the bladder radioactivity was significantly higher than the uncorrected GFR (*P* < 0.05). For obstructed kidneys, when the GFR was calculated based on the renal uptake rate at 1-2 min and 2-3 min, there was no significant difference between the GFR corrected by the bladder radioactivity and the uncorrected GFR (*P* > 0.05). Thus, based on the 1-2 min renal uptake rate calculation, the GFR calculated by the method of bladder radioactivity correction did not change the GFR results of the obstructed or unobstructed kidneys on the whole. When calculating the GFR based on the 2-3 min renal uptake rate, the unobstructed kidney GFR calculated by the bladder radioactivity correction was significantly increased but did not significantly affect the GFR of the obstructed kidney.

### 3.5. Comparison of GFR before and after the Protein Load Test

**Figure 2:**
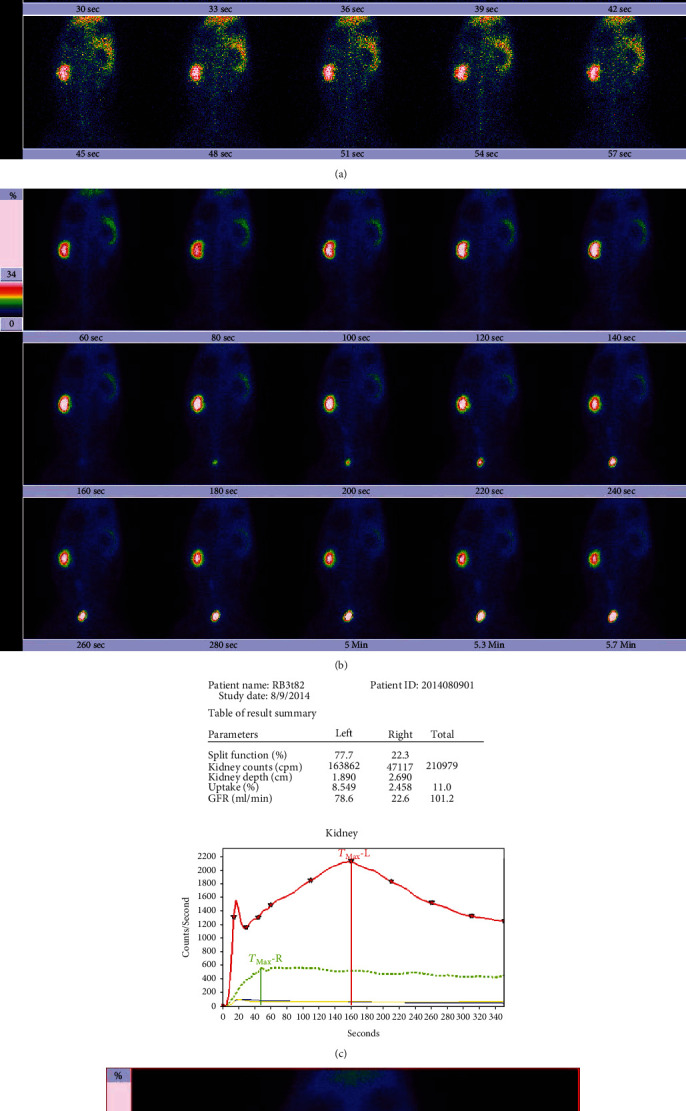
Results of dynamic renal scintigraphy at baseline in the rabbit. The examination was performed on the 82nd day of right ureteral obstruction; the time below each frame of the images in panels (a) and (b) indicates the start time of acquisition. (a) The phase of blood flow perfusion. (b) The phase of renal function. No radioactivity is observed in the bladder in 60-120 s, and a small amount of urine radioactivity distribution is observed in the bladder in 120-180 s. (c) The renogram and the functional parameters. The GFR of the left kidney is 78.6 ml/min, and the GFR of the right kidney is 22.6 ml/min. (d) The outlined ROI of the kidney and background.

**Figure 3:**
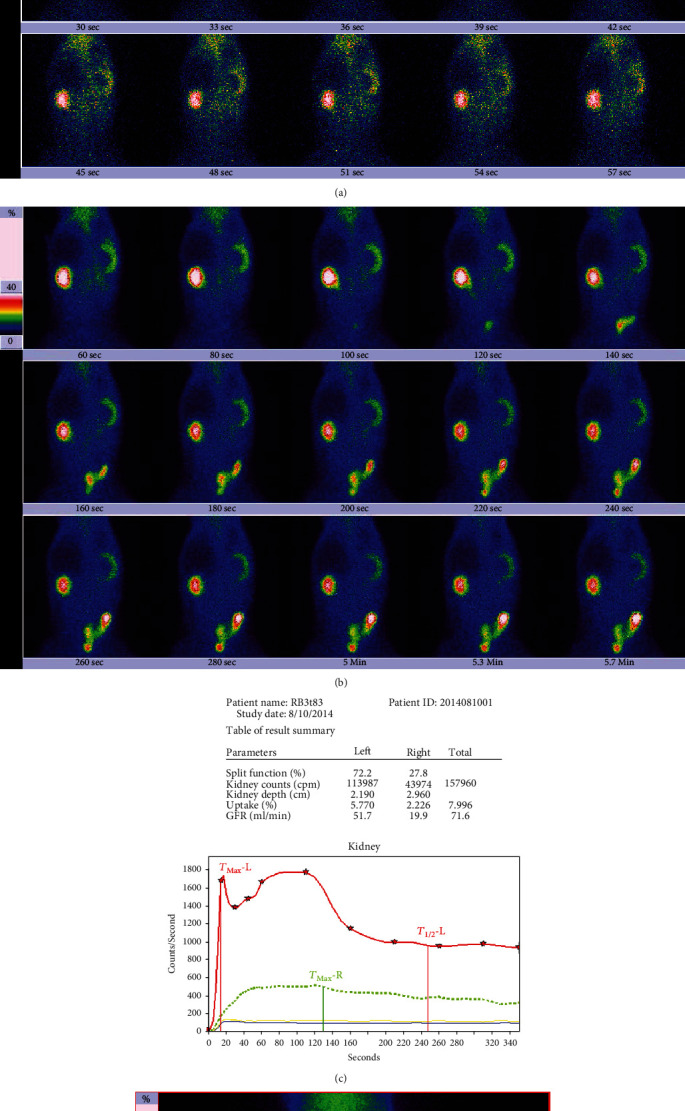
Results of dynamic renal scintigraphy in the protein load test in the rabbit. The examination was performed on the 83rd day of right ureteral obstruction; the time below each frame of the images in panels (a) and (b) indicates the start time of acquisition. (a) The phase of blood flow perfusion. (b) The phase of renal function; a small amount of urine radioactivity appeared in the bladder during 60-120 s, and much radioactivity appeared in the bladder during 120-180 s. (c) The renogram and the functional parameters. The GFR of the left kidney is 51.7 ml/min; the GFR of the right kidney is 19.9 ml/min. The left renal GFR is significantly lower than the GFR 78.6 ml/min of baseline status (Figure 2). After the renal uptake rate is corrected by bladder radioactivity, the left kidney GFR increases significantly to 88.5 ml/min, and the right kidney GFR is 20.9 ml/min. (d) The outlined ROI of the kidney and background.

The results in Table 4 are the GFR calculated based on the 2-3 min kidney uptake. For the unobstructed left kidney, most of the GFRs in the protein load test (89.5%) were higher than the baseline GFRs; after correction with the bladder radioactivity, all of the GFRs in the protein load test were higher than the baseline GFRs. For the obstructed right kidney, whether or not corrected by bladder radioactivity, there was no significant difference in the GFR between the protein load test and baseline status (*P* > 0.05). In our experiments, one rabbit was subjected to DRS at baseline (Figure 2) and in the protein load test (Figure 3). In the protein load test, a lot of urine had entered its bladder, so the load GFR of its unobstructed left kidney was greatly reduced, resulting in a significantly lower load GFR (Figure 3) than the baseline GFR (Figure 2). However, the load GFR of the left kidney was significantly increased after correction with bladder radioactivity.

## 4. Discussion

### 4.1. Gates Algorithm and Bladder Imaging Time

There are many influencing factors when measuring the split renal GFR of humans based on DRS [10–13, 23]. Therefore, the quality control of GFR measurement is very important [24–27]. The Gates method for calculating GFR is based on renal uptake at 2-3 min after injecting a radiotracer [3–6]. The reliability of the 2-3 min renal uptake rate directly affects the GFR measurements. The effect of EBI at 2-3 min on GFR was not considered in the Gates study. If the bladder is imaged at 2-3 min, it indicates that the renal urine enters the bladder and the radioactivity in the kidney is reduced. For the same patient, the GFRs calculated in the case of EBI and non-EBI will be different. In fact, it is very difficult to see a significant bladder signal during the 2-3 min period in human ^99m^Tc-DTPA DRS. According to this clinical fact, the Gates algorithm can be considered to be based on the fact that the radiotracer did not leave the kidneys, and the bladder was not imaged in the 2-3 min period [28].

### 4.2. Influence of EBI on the GFR Measurement by the Gates Method

In order to accurately determine the renal excretion or diagnose hydronephrosis using the characteristics of a renogram and DRS, we often require patients to drink enough water before imaging to avoid the urinary retention of the renal pelvis caused by dehydration from affecting the diagnosis of results [26, 29]. This measure was often necessary and appropriate for humans because we did not find that proper water drinking (5-10 ml/kg, water volume/body mass) could cause ^99m^Tc-DTPA to leave the kidney prematurely and result in a case of EBI. However, the bladder imaging time of experimental animals is different from that of humans. In the analysis of dogs, many researchers have observed the early imaging of their bladders in 3 min [8, 9, 19, 30]. Bladder imaging of white rabbits has not been reported. We observed the time of bladder imaging of adult white rabbits after ^99m^Tc-DTPA injection. We found that bladder imaging at 2-3 min appeared in most rabbits, and bladder imaging also appeared in a small part of rabbits at 1-2 min. The EBI of experimental rabbits will affect the accuracy and stability of GFR calculation by the Gates method. Studies have shown that EBI in experimental rabbits can lead to a decrease in the GFR of unobstructed kidneys, conventionally calculated, and it may also result in a decrease in the correlation between the 1-2 min uptake rate and the 2-3 min uptake rate of unobstructed kidneys as well as an erroneous phenomenon in which the GFR of unobstructed kidneys in the protein load test is lower than the GFR at baseline. In this study, EBI was not observed during 0-1 min of DRS in any adult white rabbit in the setting of adequate water supply and free drinking. Although the bladders of a few experimental rabbits were imaged at 1-2 min, the quantity of the radiotracer in the bladder was generally small. At this point, the contribution of glomerular filtration to the intravesical radiotracer was also correspondingly small. In our study, the maximum contribution of glomerular filtration to bladder radioactivity was 7 ml/min, indicating that the EBI during 1-2 min did not have a large effect on renal GFR. However, at 2-3 min after injecting the radiotracer, 57.3% of the rabbits had bladder imaging. Although the quantities of the bladder radiotracer in different rabbits were different, the contribution of glomerular filtration to the intravesical radiotracer was often larger at 2-3 min than that at 1-2 min. In this study, it was observed that the maximum contribution of glomerular filtration to bladder radioactivity was approximately 40 ml/min. Obviously, the 2-3 min bladder imaging has a greater and more extensive influence on renal GFR. Because the urine radioactivity in the early bladder imaging came from the unobstructed kidney, the EBI of experimental rabbits had an effect mainly on the GFR of the unobstructed kidney but had no significant effect on the GFR of the severely obstructed kidneys.

### 4.3. How Can We Avoid the Influence of EBI on GFR?

To avoid the influence of EBI on the GFR measurement and the evaluation of split renal function, some scholars recommend using time periods different from 2-3 min, such as 60-180 s [8, 9], 30-120 s [19, 30], 60-120 s [24, 29], and 60-150 s [24], to calculate the renal uptake rate and GFR. However, the fact that adult white rabbits presented bladder imaging at 60-120 s suggests that it is difficult to completely eliminate this influencing factor by selecting the above time periods. If the evaluation of renal function is the main purpose, to avoid the influence of EBI on the accuracy of GFR measurement by the Gates method, it is easy and effective to appropriately control the amount and time of water drinking and delay the discharge of urine into the bladder. At this time, although it is not conducive to the evaluation of renal excretion, it is very useful for the accurate measurement of GFR. In the case of severe hydronephrosis on one kidney, we consider that it is not possible that the urine in this kidney prematurely excretes into the bladder. The radioactivity that enters early into the bladder at 2-3 min should be added into the contralateral kidney, and then the GFR is recalculated. By this remedial algorithm, the effect of EBI on GFR in unobstructed kidneys can also be avoided. In this study, after being corrected by bladder radioactivity, the correlation between the 1-2 min uptake rate and the 2-3 min uptake rate of unobstructed kidneys was significantly improved; the corrected GFR of the unobstructed kidney increased to varying degrees by comparison with uncorrected GFR; at the same time, the unreasonable reduction of GFR in the protein load test in the unobstructed kidney was also corrected. In fact, in many cases, it is difficult to distinguish which kidney the urine radioactivity that enters the bladder early comes from, so this indirect remediation algorithm is often lacking in rationality. However, in animal studies of severe hydronephrosis, if the primary goal is to observe the functional change of the hydronephrotic kidney, rather than observing the unobstructed kidney, conventional GFR measurements are acceptable; so, the GFR of the severely hydronephrotic kidney does not need to be corrected by bladder radioactivity. The study by Gates showed a similar strong positive correlation between the human 1-2 min and 2-3 min renal uptake rates and the 24-hour endogenous inosine clearance rate [3]. Because the correlation between the 2-3 min renal uptake rate and the 24 h inosine clearance rate was slightly higher than that between the 1-2 min renal uptake rate and the inosine clearance rate, Gates selected the 2-3 min renal uptake rate to establish an equation calculating the GFR. Actually, the 1-2 min renal uptake rate can also be used to establish an equation calculating the renal GFR, except that the coefficient and constant terms of the equation will be different from those in the equation of 2-3 min. For adult white rabbits, when drinking enough water in a natural state, the probability of EBI at 1-2 min after injecting the radiotracer is very low, and the urine radioactivity is very small; thus, the effect of bladder radioactivity on renal GFR is not significant. Therefore, using the 1-2 min renal uptake rate to establish the equation of GFR calculation is more suitable for the evaluation of renal function in adult white rabbits and is more conducive to weakening or avoiding the effect of EBI on GFR results. However, in the Gates method, the relevant parameters of the GFR calculation equation were not established by the 1-2 min renal uptake rate, so we have no ready-made calculation equations to apply. If we apply the renal uptake rate of 1-2 min directly to the Gates equation, the result will be different from that by the established GFR calculation equation based on the 1-2 min renal uptake rate, leading to systematic deviations. Even so, the GFR based on the 1-2 min uptake rate is still more stable than that based on the 2-3 min uptake rate. Our study shows that when the renal uptake rate of 1-2 min was directly applied to the Gates equation to calculate the GFR, the difference between the GFR corrected by bladder radioactivity and the uncorrected GFR was not statistically significant. Therefore, calculating the GFR by applying the 1-2 min renal uptake rate directly to the Gates equation can also attenuate or avoid the effects of EBI on the stability of GFR results.

## 5. Conclusion

In DRS, EBI of experimental rabbits is very common. Bladder imaging during 2-3 min necessarily affects the accuracy of the Gates algorithm for calculating GFR based on the 2-3 min renal uptake rate. This study shows that adequate hydration is not conducive to the accurate measurement of renal GFR in experimental rabbits by the Gates method, and the proper control of water drinking in rabbits for avoiding EBI is conducive to accurate measurement of GFR. In addition, calculating the GFR by the renal uptake rate at 1-2 min may also help to reduce or avoid the effects of EBI on GFR measurements.

## Figures and Tables

**Table 1:** Comparison of bladder radiotracer uptake and its contribution from renal filtration between 1-2 min and 2-3 min after injection.

Bladder radiotracer	Unit	1-2 min				2-3 min				*t*	*P*
		*n*	Minimum	Maximum	Mean ± SD	*n*	Minimum	Maximum	Mean ± SD		
Uptake rate	%	12	0.20	0.80	0.48 ± 0.14	68	0.14	4.04	1.13 ± 0.81	2.800	0.006
Contribution from total renal filtration	ml/min	12	1.81	7.37	4.27 ± 1.33	68	1.21	37.20	10.32 ± 7.35	2.830	0.006
Contribution from left renal filtration	ml/min	12	1.81	7.37	3.71 ± 1.59	68	1.11	37.20	9.28 ± 7.02	2.723	0.008
Contribution from right renal filtration	ml/min	3	2.10	2.35	2.23 ± 0.13	12	1.11	12.94	5.90 ± 3.14	4.029	0.002

Notes: GFR—glomerular filtration rate; SD—standard deviation; *n*—number of dynamic renal scintigraphy scans presenting the entire bladder in the field of view and having bladder radiotracer uptake in 1-2 min or 2-3 min.

**Table 2:** Correlation between the renal uptake rate at 1-2 min and that at 2-3 min after injecting the radiotracer.

Obstruction	Uptake rate	Uncorrected by bladder radioactivity			Corrected by bladder radioactivity			Correlation improvement after correction
		*n*_1_	*r*	*P*	*n*_2_	*r*	*P*	
No obstruction	Total kidney	40	0.911	<0.001	35	0.974	<0.001	Improved
	Left kidney	40	0.865	<0.001	35	0.952	<0.001	Improved
	Right kidney	40	0.936	<0.001	35	0.967	<0.001	Improved

Right ureteral obstruction	Total kidney	171	0.907	<0.001	123	0.975	<0.001	Improved
	Left kidney	171	0.859	<0.001	123	0.979	<0.001	Improved
	Right kidney	171	0.967	<0.001	123	0.968	<0.001	No improvement

Notes: *n*_1_—number of dynamic renal scintigraphy scans presenting the partial or entire bladder in the field of view; *n*_2_—number of dynamic renal scintigraphy scans presenting the partial or entire bladder in the field of view and having no bladder radiotracer uptake at 1-2 min or 2-3 min plus those presenting the entire bladder in the field of view and having bladder-tracer uptake at 1-2 min or 2-3 min.

**Table 3:** Comparison between renal GFR corrected by bladder radioactivity and uncorrected renal GFR.

Obstruction	Kidney	GFR based on 1-2 min (ml/min)					GFR based on 2-3 min (ml/min)				
		*n*_1_	Uncorrected	Corrected	*t*	*P*	*n*_2_	Uncorrected	Corrected	*t*	*P*
No obstruction	Total	40	71.1 ± 19.0	71.4 ± 19.0	1.776	0.084	35	81.8 ± 22.7	86.5 ± 21.8	3.421	0.002
	Left	40	38.7 ± 9.5	38.8 ± 9.4	1.776	0.084	35	43.9 ± 11.5	46.4 ± 10.9	3.340	0.002
	Right	40	32.4 ± 10.4	32.6 ± 10.4	1.776	0.084	35	37.9 ± 12.1	40.2 ± 11.7	3.489	0.001

Right ureteral obstruction	Total	165	71.6 ± 20.1	71.8 ± 20.1	1.803	0.073	123	81.0 ± 23.7	85.9 ± 23.2	7.004	<0.001
	Left	165	49.4 ± 14.4	49.6 ± 14.5	1.842	0.067	123	58.7 ± 17.4	63.6 ± 17.4	7.001	<0.001
	Right	165	22.3 ± 11.6	22.3 ± 11.6	1.000	0.319	123	22.3 ± 13.3	22.3 ± 13.3	1.000	0.319

Notes: GFR—glomerular filtration rate; *n*_1_—number of dynamic renal scintigraphy scans presenting the partial or entire bladder in the field of view and having no bladder radiotracer uptake in 1-2 min plus those presenting the entire bladder in the field of view and having bladder radiotracer uptake at 1-2 min; *n*_2_—number of dynamic renal scintigraphy scans presenting the partial or entire bladder in the field of view and having no bladder radiotracer uptake at 2-3 min plus those presenting the entire bladder in the field of view and having bladder radiotracer uptake in 2-3 min.

**Table 4:** Comparison of GFR based on 2-3 min renal uptake between baseline status and protein load test.

Kidney	Uncorrected GFR (*n* = 19)					Corrected GFR (*n* = 19)				
	Baseline*^a^*	In PLT*^b^*	*b* < *a*	*t*	*P*	Baseline*^c^*	In PLT*^d^*	*d* < *c*	*t*	*P*
Total	79.8 ± 20.8	99.4 ± 27.3	10.5% (2/19)	3.731	0.002	81.9 ± 19.7	107.2 ± 22.9	0.0% (0/19)	6.711	<0.001
Left	53.2 ± 14.7	68.9 ± 15.3	10.5% (2/19)	3.823	0.001	54.6 ± 14.1	76.2 ± 13.7	0.0% (0/19)	8.846	<0.001
Right	26.6 ± 16.1	30.5 ± 20.1	36.8% (7/19)	1.612	0.124	27.3 ± 16.4	31.0 ± 20.6	36.8% (7/19)	1.553	0.138

Notes: GFR—glomerular filtration rate; PLT—protein load test; *n*—paired number of dynamic renal scintigraphy between GFR at baseline and GFR in PLT.

## Data Availability

The data in this study are available from the corresponding author (Changyin Wang) on reasonable request.
